# Talilmogene Iaherparepvec generates systemic T-cell-mediated anti-tumor immunity

**DOI:** 10.1186/2051-1426-1-S1-P198

**Published:** 2013-11-07

**Authors:** Julia Piasecki, Le Tiep, Jean Zhou, Courtney Beers

**Affiliations:** 1 Amgen, Seattle, WA, USA

## 

Talimogene laherparepvec is an investigational oncolytic immunotherapy based on a modified herpes simplex virus type-1 (HSV-1) that is designed to selectively replicate in tumor tissue and to stimulate a systemic antitumor immune response. Intralesional administration of talimogene laherparepvec is intended to result in oncolysis within injected tumors. Iterative viral replication of virus within permissive tumor tissue results in lytic cell destruction and local release of progeny virus and cellular derived antigens. GM-CSF, a product of the viral transgene, is also produced locally such that it can recruit and stimulate antigen presenting cells which, in addition to relevant tumor-derived antigens, are required for the initiation of a systemic antitumor immune response. A phase 3 clinical trial of talimogene laherparepvec in regionally or distantly metastatic melanoma has recently reported a 26% ORR objective response rate (6% for GM-CSF alone){(J Clin Oncol 31, 2013 (suppl; abstr LBA9008)}. Since the immune mediated anti-tumor mechanisms have yet to be fully understood, we sought to identify immune-specific changes post talimogene laherparepvec administration in the A20 syngeneic contralateral tumor model. We analyzed immune populations in both injected and non-injected tumors and in peripheral hematopoietic tissues at 2, 5 and 10 days post injection. Animals received one 5x10^6^ PFU intratumoral dose of talimogene laherparepvec which induces complete regression in approximately 70-100% of the injected tumors and approximately 50-60% of the contralateral non-injected tumors. Injection of virus resulted in marked increases in injected tumor draining lymph node and spleen size compared to control animals. Upon further flow cytometric analysis, changes in T-cell populations were observed in the injected and non-injected tumor draining lymph nodes, spleen and peripheral blood and T-cells expressed higher levels of cell surface activation markers as early as 2 days post-treatment in talimogene laherparepvec treated animals as compared to vehicle control animals. To functionally examine the T-cell-mediated anti-tumor immunity, we evaluated the memory response by re-challenging mice that completely regressed their primary A20 tumors with either A20 or three other Balb/c-derived tumor cell lines; 4T1, CT26 and RENCA. Interestingly, the mice displayed broad immunity to Balb/c tumor cells and rejected the A20, CT26 and RENCA but not 4T1 tumor cells whereas all the tumor cell lines grew robustly in control naïve animals. In addition, splenic T-cells from talimogene laherparepvec-treated mice induced A20 tumor cell killing ex vivo while T-cells from vehicle control-treated mice did not. Intratumoral injection of talimogene laherparepvec systemically activates T cells and generates anti-tumor immunity in mice.

